# Construction and Preliminary Validation of a Multidimensional Physical Health Assessment System for Preschool Children in China

**DOI:** 10.3390/bs16101925

**Published:** 2026-10-17

**Authors:** Xinge Tan, Xiaowei Li, Bingcheng Gao

**Affiliations:** 1 Faculty of Education, Beijing Normal University, Beijing 100875, China; 202531010038@mail.bnu.edu.cn; 2 China National Academy of Educational Sciences, Beijing 100088, China

**Keywords:** physical health, preschool children, assessment system, health promotion, Analytic Hierarchy Process

## Abstract

Early childhood is the foundational stage of lifelong health management and is associated with long-term physical and developmental outcomes. Yet health problems are emerging at increasingly younger ages, while existing assessment systems remain fragmented, typically emphasizing motor performance while relegating clinical health indicators. This study developed an assessment system specified as a formative composite index, incorporating clinical health indicators across three core dimensions: body morphology, physical fitness, and clinical health, comprising 13 indicators weighted using the Analytic Hierarchy Process. The index was evaluated in 1462 children aged 3–7 years in Shandong Province, China. The weighting scheme showed good consistency, and the indicators exhibited low multicollinearity. Structural validity was evaluated using a MIMIC model, and deletion sensitivity analyses indicated general stability across indicators. Overall, the results provide preliminary validation for the assessment system; further refinement and broader validation are needed.

## 1. Introduction

Early childhood is a critical period for growth and development, serving as the foundation for lifelong physical health (Zhao et al., 2024). Physical health during this stage carries significant implications for subsequent health and developmental pathways. For example, childhood obesity has been associated with cardiovascular disease (Cieżki et al., 2024), sleep disorders (Inja & Cielo, 2025), and poorer social and executive function (Pearce et al., 2023). However, recent evidence suggests that these associations are not entirely consistent in preschool children. A cross-sectional study found no significant differences across BMI categories in movement behaviours, motor skills, or executive functions (Ltifi et al., 2026), while another study found only limited associations between BMI and 24 h movement behaviours and executive functions (Meksi et al., 2026). Physical fitness during this period has also been linked to later cognitive and academic outcomes, including executive function, reading and mathematics ability, and academic achievement (Fernandes et al., 2024; Z. Sun et al., 2024). Intervention evidence further suggests that physical activity can improve motor skills and inhibitory control in preschoolers (Ltifi et al., 2025).

However, health problems are emerging at increasingly younger ages, with excess weight, low fitness, dental caries, visual impairment, nutritional deficiencies, and anaemia frequently co-occurring during this period. Recent studies have documented declining or inadequate physical activity and fitness levels among preschool children (Haugland et al., 2024; Bourke et al., 2023). For example, in a pilot study of 112 preschoolers, only 18% met all three WHO movement recommendations, while 41% met the physical activity recommendation, 53% met the screen time recommendation, and 81% met the sleep duration recommendation (Ltifi et al., 2024). These findings raise concerns about motor development and long-term health. Clinical health problems are also common in this age group. Dental caries remains highly prevalent (Chen et al., 2024), and visual impairments are increasingly reported (Shen et al., 2025; Y. Sun et al., 2025). Anaemia is also relevant in this age group, and national guidance specifies how it should be screened (National Health Commission of the People’s Republic of China, 2013). Overweight and obesity remain a major global concern (Bhutta et al., 2023), with prevalence rising steadily from 1990 to 2021 and projected to increase further through 2030 (Zhang et al., 2024). These findings highlight the need for early detection and systematic monitoring of preschool children’s physical health.

Traditionally, early childhood health evaluation has centered on two primary, interrelated domains: body morphology and physical fitness. Associations of body morphology (height, weight, and BMI) with physical fitness and motor performance have been reported in early childhood (Rico-González et al., 2024). Specifically, body morphology reflects physical growth and nutritional status, whereas physical fitness reflects functional capacities such as cardiorespiratory fitness, muscular strength, flexibility, balance, and motor proficiency. The assessment of body morphology and physical fitness has shifted from measuring isolated physical attributes to multiple dimensions. Widely recognized assessment batteries are the European Test of Physical Fitness (EUROFIT) (Mupondi et al., 2024), FitnessGram (Day et al., 2023; Welk & Meredith, 2011), and the Assessing Levels of Physical Activity health-related fitness test battery (ALPHA Fitness) (Ruiz et al., 2011). However, these systems were developed mainly for school-aged children; researchers subsequently designed preschool-specific instruments. The Assessing FITness in PREschoolers (PREFIT) battery was designed for preschool children to evaluate body composition, cardiorespiratory endurance, musculoskeletal fitness, and motor skill competence (Soto-Sánchez et al., 2026).

However, physical fitness and body morphology alone do not constitute children’s overall physical health. Overall physical health is composed of physical growth, functional capacity, and clinically identifiable health conditions (World Health Organization & United Nations Children’s Fund, 2023); clinical health is therefore a constitutive component of the construct. These clinical health indicators, such as dental caries, visual impairment, malnutrition, and anaemia, are generally assessed through medical screening systems rather than integrated into fitness batteries (Amade et al., 2023). In some cases, a child may demonstrate adequate physical fitness in a single assessment while still experiencing underlying health risks. These considerations underscore the need to treat clinical health as a constitutive component of overall physical health when assessing children’s health.

The separation between body morphology, physical fitness and clinical health assessment is also evident in China. Current preschool physical evaluations are conducted predominantly within early childhood education settings, whereas health indicators such as visual acuity are captured through isolated public health screening procedures (Y. Sun et al., 2025). Consequently, body morphology, physical fitness, and clinical health remain distinct components rather than parts of an integrated assessment framework. Comprehensive evaluation of developmental level for children and adolescents (GB/T 31178–2014) integrates clinical health and mental health into a composite evaluation for children and adolescents aged 7–18 years (National Health Commission of the People’s Republic of China, 2014). This standard treats these domains as constituent components of developmental level, and provides a precedent for integrating clinical health indicators with fitness measures in a composite.

Building on this precedent, preschool physical health can be approached not as a single underlying trait but as a multidimensional composite assessment encompassing several distinct domains. Within such a composite, different indicators contribute distinct aspects of the condition being assessed rather than simply reflecting the same underlying attribute. In the present study, body morphology captures physical growth and body composition, physical fitness captures functional physical capacity, and clinical health captures clinically identifiable conditions and health risks. These domains provide complementary information that cannot be adequately represented by any single dimension. For example, a child may be physically fit yet anaemic, or have normal body morphology yet untreated dental caries. Omitting any one of these domains would therefore narrow the scope of the assessment. The use of multiple indicators to capture different aspects of a broader health condition is also reflected in established composite measures, including the Apgar score and frailty indices (Apgar, 1953; Rockwood & Mitnitski, 2007).

The present study developed an assessment system for preschool children’s physical health, specified as a formative composite index constituted by body morphology, physical fitness, and clinical health. This study therefore aimed to (i) construct this assessment system and (ii) evaluate the indicator weights, provide initial validation evidence, and conduct a preliminary application of the system.

## 2. Methods

### 2.1. Construction of the Physical Health Assessment System for Preschool Children

#### 2.1.1. Indicator Selection

Indicators for body morphology and physical fitness were drawn from the National physical fitness measurement standard (National Physical Fitness Monitoring Center of China, 2023). Candidate indicators for clinical health were identified from the Measures for the health care management of nurseries and kindergartens (Order No. 76) (Ministry of Health of the People’s Republic of China & Ministry of Education of the People’s Republic of China, 2010). The initial indicator pool encompassed dental caries, overweight/obesity, refractive error, malnutrition, anaemia, abnormal spinal curvature, organic diseases (e.g., cardiac, hepatic, and renal conditions), and routine physical examination parameters (e.g., ENT, musculoskeletal status, and ALT levels). Each indicator was evaluated against four predetermined criteria: (i) documented relevance to long-term child health and development; (ii) availability of age-appropriate normative standards; (iii) feasibility and acceptability of noninvasive kindergarten screening; and (iv) potential for early prevention or intervention.

Five clinical health indicators met all four criteria and were retained: dental caries, overweight/obesity, refractive error, malnutrition, and anaemia. Their public health relevance was corroborated by a regional monitoring dataset from Shandong Province (*N* = 5846; Zibo Preschool Child Health Screening Database; 2025; stratified cluster sampling): dental caries (83.2%), overweight/obesity (23.3%), refractive error (10.0%), malnutrition (6.1%), and abnormal haemoglobin (5.7%, as an indicator of anaemia).

The remaining indicators were excluded for failing to meet one or more criteria. Descriptive physical exam items (e.g., dermatological, ENT, musculoskeletal status) lacked age-specific normative scoring (criterion ii). ALT was excluded due to the invasiveness of blood sampling for routine kindergarten screening (criteria i and iii). Abnormal spinal curvature lacked preschool-specific screening norms (criterion ii). Finally, organic diseases were excluded as their diagnosis requires comprehensive clinical evaluation beyond ECE-based screening (criterion iii) and lacks potential for early modification (criterion iv).

#### 2.1.2. Determination of Indicator Weights

Because the assessment system uses a formative model, indicator weights cannot be calculated from the correlations between indicators. In addition, clinical health has often been underrepresented in existing preschool assessments, and giving it greater weight is a substantive judgment rather than an empirical one. The Analytic Hierarchy Process (AHP) was therefore used to determine the weights. AHP allows experts to compare the importance of dimensions in pairs, turning their judgments into quantitative weights with a built-in consistency check to reduce bias (Min et al., 2024). The procedure followed standard guidelines (Frish et al., 2025):

Firstly, a paired comparison judgment matrix was constructed. Eight experts across medicine, early childhood education, and higher education were invited. All experts had over five years of experience, direct involvement in child health or physical education, and familiarity with national assessment standards. Using Saaty’s 1–9 scale (Frish et al., 2025), they independently scored the relative importance of indicator pairs. While individual judgments showed moderate rank-order agreement due to diverse professional backgrounds (Kendall’s *W* = 0.40, *p* = 0.040), the aggregated weight vector showed high inter-rater reliability (ICC(2, k) = 0.93). The judgment matrix scores of the eight experts were then averaged (Table 1).

Second, indicator weights were calculated using the sum–product method. Each column of the judgment matrix was normalized according to Formula (1); the results are shown in Table 2. Then, rows were added according to Formula (2) to calculate the characteristic vector: *N* (body shape) = 0.49, *N* (physical fitness) = 0.95, *N* (clinical health) = 1.56. Finally, the average of each row was calculated according to Formula (3) to obtain the weights: *w* (body morphology) = 0.163, *w* (physical fitness) = 0.317, *w* (clinical health) = 0.520.

*a*′*ij* = *a_ij_*/Σ*_i_*_=1_*^n^ a_ij_* (1)

*N_i_* = Σ *a*′*_ij_* (*i*, *j* = 1, 2, …, *n*) (2)

*w_i_* = *N_i_*/*n* (3)

Thirdly, a consistency check was performed. The maximum eigenvalue (*λmax*) and consistency index (*CI*) corresponding to the judgment matrix were calculated according to Formula (4); the results are shown in Table 3.

*λmax* = (1/*n*) Σ [(*A·w*)*_i_*/*w_i_*];   *CI* = (*λmax* − *n*)/(*n* − 1)   (*A* = *judgment matrix*; *w* = *weight vector*; *n* = *matrix order*) (4)

The CI value was compared with the random consistency index (*RI*) (Table 4), and the *CR* value was obtained according to Formula (5). A *CR* value below 0.1 indicates acceptable consistency. In this study, *CR* = 0.03 < 0.1, indicating that the judgment matrix passed the consistency check.

*CR* = *CI*/*RI* (5)

Fourthly, indicator weights were synthesized. First-level weights were derived directly from AHP expert scores. Second-level weights for body morphology and physical fitness were calculated based on the National physical fitness measurement standard. In contrast, the clinical health dimension used a deduction-based scoring system rather than proportional weights, following the Comprehensive evaluation of development level for children and adolescents (National Health Commission of the People’s Republic of China, 2014). Because these five clinical indicators represent health risks rather than continuous performance, each child started with a baseline score of 100, with points deducted for identified health issues (Table 5). The final weights are summarized in Table 6. To test sensitivity to the aggregation method, expert judgments were also re-aggregated using the geometric mean instead of the arithmetic mean. The resulting weights differed by at most 0.0003, and the composite scores were identical (*r* = 1.000), confirming that the weighting scheme was robust.

#### 2.1.3. Measurement Model Specification

The assessment system was specified as a formative composite index, where the indicators jointly define the overall construct rather than reflecting an underlying latent trait (Jarvis et al., 2003). Because formative indicators represent distinct, non-redundant aspects of physical health, traditional internal consistency metrics are conceptually inappropriate (Hanafiah, 2020). Instead, validity was evaluated across four core phases recommended by Diamantopoulos and Winklhofer (2001): content specification, indicator specification, indicator collinearity, and external validity.

### 2.2. Validation of the Indicator System

#### 2.2.1. Participants

This cross-sectional study was conducted in Zibo, Shandong Province, between May and July 2025. Ten early childhood education (ECE) institutions participated, including six public and four private, all located in urban districts. Children were eligible if they were aged 3–7 years, had obtained parental consent, completed both the health examination and physical fitness testing, and had no severe congenital or acute illness.

The sample size was determined by the precision required to estimate the prevalence of the health conditions under study. The required number of children was calculated as follows. With *d* = 0.03 and the most conservative assumption *P* = 0.50, the minimum required sample was 1067 children.

$$n=\frac{Z_{1-\alpha /2}^{2}\times P\times (1-P)}{d^{2}}$$

Of 1672 children invited, 1510 completed assessments (162 absent due to minor illness). After excluding 48 incomplete or invalid records, the final analytic sample comprised 1462 children (87.4% overall retention). The final sample had a mean age of 5.5 years (*SD* = 0.9) and included 774 boys (52.9%) and 688 girls (47.1%). Participants were grouped according to ECE class level rather than chronological age: junior class (*n* = 246, *M* = 4.38 years, *SD* = 0.56), middle class (*n* = 517, *M* = 5.19 years, *SD* = 0.50), and senior class (*n* = 699, *M* = 6.20 years, *SD* = 0.52).

#### 2.2.2. Procedure

On-site clinical assessments were conducted by five physicians from Linzi Maternal and Child Health Hospital, Shandong Province. Before data collection, all assessors (including the five physicians and kindergarten teachers conducting physical fitness tests) completed a one-week standardized training program based on the National physical fitness measurement standard.

Height and weight were measured using a stadiometer and calibrated electronic scale to the nearest 0.1 cm and 0.1 kg, respectively, with children wearing light indoor clothing and no shoes. BMI was derived from these measurements, and overweight/obesity was classified according to the Growth standards for children under 7 years of age (WS/T 423–2022). Dental caries was evaluated during routine examinations via visual and tactile inspection, recording the number of decayed teeth. Refractive status was measured with a portable autorefractor under non-cycloplegic conditions, recording spherical and cylindrical powers in diopters to apply Table 5 criteria. Haemoglobin levels were determined from capillary blood samples using a portable analyzer.

#### 2.2.3. Statistical Analysis

Norm-referenced standard scores. Height, BMI, and the six physical fitness metrics were converted into standard scores based on sex- and age-specific norm tables from the National physical fitness measurement standard. Raw test values were mapped onto a 13-point scale (10, 30, 50, 55, 60, 65, 70, 75, 80, 85, 90, 95, and 100). BMI was scored across four categories: thin (60 points), normal weight (100 points), overweight (60 points), and obese (20 points). These scores reflect a child’s status relative to the national reference population of the same age and sex.

Criterion-referenced clinical score. Clinical health was scored via criterion-referenced evaluation. Starting from a baseline of 100 points, deductions were applied for each confirmed health condition according to Table 5. Total scores were converted to a standard scale (50–90) following the Comprehensive evaluation of development level for children and adolescents: 100 points corresponded to 90 (excellent); 97–99 corresponded to 80 (good); 87–96 corresponded to 70 (moderate); 80–86 corresponded to 60 (poor); and ≤79 corresponded to 50 (very poor).

Dimension and composite score. The three first-level dimensions were computed as weighted sums of their constituent standard scores. The first-level weights were derived from the AHP, while the second-level weights followed the National physical fitness measurement standard. The composite physical health index was formed as the weighted sum of the three dimensions.

Because raw scale units differ (morphology and fitness are norm-referenced standard scores, whereas clinical health is a criterion-referenced deduction score), dimension scores were used strictly to assess within-dimension variation rather than for direct cross-dimension comparisons.

## 3. Results

### 3.1. Validity of the Formative Index

Following Diamantopoulos and Winklhofer (2001), the evidence for the index is reported in the order of content specification, indicator specification, indicator collinearity, and external validity. The first two are detailed in Section 2.1 and are not repeated here. The following sections address indicator collinearity and external validity, followed by two further checks: structural validity and robustness to indicator removal.

Indicator collinearity was evaluated to ensure that formative indicators provided distinct, non-redundant information. Variance inflation factors (VIFs) were calculated both within each dimension and across the pooled set of indicators. All VIF values were well below the conservative threshold of 3.3 recommended for formative measures (Petter et al., 2007). Specifically, VIFs within individual dimensions did not exceed 1.31. In the pooled set, all VIFs remained below 1.5, with the sole exception of BMI (2.94) and overweight/obesity status (2.90); these two indicators are mathematically linked by definition (*r* = −0.80). Inter-item correlations further confirmed low redundancy: 74 of the remaining 78 indicator pairs exhibited |*r*| < 0.30, and the mean correlation within the clinical dimension was 0.01. Overall, these results demonstrate that the formative indicators contribute largely unique information.

External validity was evaluated using a four-item parent-reported scale (Cronbach’s α = 0.74) assessing child general health (relative health, doctor visit frequency, expected health status, and cold frequency). As detailed in Table 7, the formative index correlated highly with the overall criterion (*r* = 0.79, *p* < 0.001), outperforming the conventional assessment score that comprises only physical fitness and body morphology (*r* = 0.53). At the dimension level, the correlations were 0.60 for clinical health, 0.42 for body morphology and 0.40 for physical fitness. Furthermore, the formative index added substantial explained variance over the conventional assessment score (Δ*R*^2^ = 0.35; *R*^2^ from 0.28 to 0.63). These findings demonstrate that the proposed index captures critical clinical health information omitted by traditional physical-only assessments.

Given the formative nature of the index, structural validity was evaluated via a MIMIC model, where the 13 indicators predict a latent physical health variable reflectively measured by four parent-reported items. The MIMIC model showed good fit: *χ*^2^(41) = 27.95, *p* = 0.940, RMSEA = 0.000, CFI = 1.000, SRMR = 0.013, but this should be read alongside the strong zero-inflation of the clinical indicators and the use of MLR estimation. The 13 indicators explained 76.2% of the variance in the composite index (*R*^2^ = 0.762). As presented in Table 8, 12 of the 13 indicators demonstrated statistically significant structural coefficients (*γ*). The largest standardized structural coefficients were observed for dental caries (*γ* = −0.449, *p* < 0.001) and overweight/obesity (*γ* = −0.386, *p* < 0.001) within the clinical dimension, height (*γ* = 0.342, *p* < 0.001) in body morphology, and balance-beam walking (*γ* = 0.210, *p* < 0.001) in physical fitness. Anaemia was the only non-significant indicator (*γ* = −0.028, *p* = 0.139). Nevertheless, anaemia showed no statistically significant effect, primarily because its low prevalence (only 1.0% of the sample) restricted statistical power. Despite this, it was retained on conceptual grounds as a mandatory component of national early health screening standards. As detailed in Table S1, sensitivity analyses confirmed the robustness of these coefficients across alternative estimators, binary codings, and bootstrapping.

Robustness of the composite metric was examined using a deletion counterfactual to assess the sensitivity of the composite to individual indicators. Each indicator was removed in turn, its weight within the corresponding dimension was redistributed among the remaining indicators, and the composite was recomputed (Table S2). The composite was highly stable under most deletions: removing any one of the six fitness tests yielded correlations with the full composite of *r* ≥ 0.977, while removing refractive error, malnutrition, or anaemia yielded *r* ≥ 0.986. Larger changes were observed after removing height (*r* = 0.898), overweight/obesity (*r* = 0.888), or dental caries (*r* = 0.820). The largest change occurred when BMI and overweight/obesity were removed simultaneously (*r* = 0.768), consistent with their shared anthropometric basis. Overall, the composite showed limited sensitivity to the deletion of individual indicators.

### 3.2. Preliminary Application of the System

The children’s scores on the indicators were as follows. At the indicator level, 564 children (38.6%) scored below 60 points (the lower bound of the pass band in the national scoring system) on height; 872 (59.6%) on sit-and-reach; 570 (39.0%) on the standing long jump; 394 (27.0%) on grip strength; 570 (39.0%) on the continuous two-foot jump; 470 (32.2%) on the 15 m obstacle run; and 946 (64.7%) on the balance beam. The proportion of children scoring below the national criterion was highest for the balance beam and sit-and-reach, and lowest for grip strength.

In clinical health, 1025 children (70.1%) had dental caries, 87 (6.0%) were obese and 121 (8.3%) were overweight, 97 (6.6%) had refractive abnormalities, 53 (3.6%) had malnutrition, and 14 (1.0%) had anaemia. Dental caries was by far the most prevalent condition, affecting seven children in ten.

## 4. Discussion

### 4.1. Rationality of the Physical Health Assessment System for Preschool Children

The proposed assessment system specifies preschool physical health as a formative composite of body morphology, physical fitness, and clinical health, with clinical health included as a constitutive component rather than as an addition to a fitness-based measure. The present results provide preliminary support for this system across the four aspects examined. First, the content was derived from national standards, yielding 13 indicators across three dimensions. Second, the weighting scheme showed good consistency (*CR* = 0.03). Third, indicator collinearity was low (maximum VIF = 2.94), confirming non-redundancy. Fourth, the index correlated strongly with parent-reported child health (*r* = 0.79), outperforming the conventional physical-only assessment (*r* = 0.53) and substantially increasing explained variance (from 28% to 63%). Structural validity was further supported by a MIMIC model (12 of 13 indicators significant), with robustness confirmed across sensitivity analyses. Overall, these findings provide preliminary evidence, given the parent-reported criterion, cross-sectional design, and lack of cross-validation.

Furthermore, the convergence between expert-derived weights and data-driven validity estimates supports the structural rationality of the index. Clinical health received both the highest AHP weight (0.520) and the strongest empirical association with parent-reported health (*r* = 0.60), followed by physical fitness and body morphology–a ranking mirrored in the MIMIC model (*γ* = 0.66, 0.47 and 0.33). Although this association partly reflects content overlap with criterion items (for example, items asking whether the child needs to see a doctor or has reached the expected health status), it reinforces the primary weight assigned to clinical risks. Moreover, the literature confirms that body morphology, physical fitness, and clinical conditions interact in early childhood: weight status influences motor performance (Podogrodzki et al., 2025; Dubale et al., 2025), visual status relates to morphology (Liu et al., 2024), and dental caries co-occurs with obesity (Elkhodary et al., 2024). These interrelationships demonstrate that the three domains are complementary, justifying their integration into a single assessment system.

From a practical perspective, integrating clinical health unifies previously fragmented screening procedures into a single monitoring tool. The system is designed for kindergarten and regional-level health monitoring and early risk identification, rather than clinical diagnosis. Such integration facilitates early detection during critical developmental windows. In practice, therefore, the composite index should always be reported together with its three dimensions, so that specific health risks can be identified and acted upon.

### 4.2. Current Physical Health Status of Preschool Children

Applying the assessment system at the indicator and clinical levels showed that the children’s physical health profile was uneven across the three domains. Dental caries was by far the most common condition, affecting seven children in ten, whereas overweight and obesity together affected about one in seven and each remaining clinical condition affected fewer than one in fifteen. In physical fitness, shortfalls relative to the national criterion were concentrated in flexibility and dynamic balance, on which roughly six children in ten fell below the passing standard, by contrast with grip strength, on which about a quarter did.

Two features of this profile are worth noting. First, the shortfalls are not concentrated in a single domain: the most prevalent problem was a clinical condition, whereas the largest fitness shortfalls occurred in flexibility, dynamic balance and lower-limb coordination rather than in muscular strength. Second, the clinical conditions that were most common are precisely those whose symptoms are often not obvious in early childhood and therefore depend on screening to be detected. Taken together, these results suggest that although serious disease was rare, the children’s physical health reserves were limited, and that the limitations differed by domain. The high prevalence of dental caries is consistent with recent evidence. A cross-sectional study in Huizhou reported a marked age-related rise in caries prevalence among preschool children, from 58.2% at age 3 to 80.5% at age 5 (Chen et al., 2024), and a meta-analysis confirms that dental caries remains a widespread burden among preschool children worldwide (He et al., 2024). Likewise, the overweight and obesity findings are consistent with recent Chinese evidence showing a substantial and rising burden of early childhood overweight (18.4%) and obesity (10.3%) (Li et al., 2025).

## 5. Limitations and Future Directions

This study provides initial evidence for a preschool physical health assessment system, but several limitations warrant consideration.

First, study design and generalizability were constrained. The cross-sectional design at a single time point precluded evaluating test–retest reliability, temporal stability, or long-term predictive validity for child outcomes. Additionally, the sample was recruited via convenience sampling from urban kindergartens in a single city, and no rural or township kindergartens were included, limiting the generalizability of the reported prevalence rates to rural or broader national populations. Multicenter longitudinal studies using stratified probability sampling are needed.

Second, external validation remains preliminary, with potential circularity and shared method variance. Validation relied on parent-reported health ratings rather than objective clinical criteria or independent cohorts. Because parent reports assessed doctor visits and illness frequency, content overlap with clinical health indicators introduces potential circularity in construct validation. Lack of out-of-sample external validation in independent cohorts further limits conclusions regarding diagnostic utility. Future research should apply objective clinical diagnoses and validate the composite across independent populations.

Third, assessor reliability was not formally quantified. The clinical indicators were obtained during routine health examinations performed by five physicians, and the physical fitness tests were conducted by kindergarten teachers; all assessors completed the same one-week standardised training programme, but no inter-rater reliability study was carried out, and no repeated measurements were taken within the same child. The consistency of the anthropometric, fitness, refractive and haemoglobin measurements across assessors therefore remains unquantified. Such measurement error would be expected to attenuate rather than inflate the associations reported in this study. Future studies using research-specific measurement protocols should report inter-rater reliability for each measurement domain.

Fourth, the weighting scheme and structural modeling remain preliminary. Indicator weights were derived via expert-based AHP rather than data-driven estimation, preventing stability tests across samples. Redundancy exists between mathematically linked metrics (BMI and overweight/obesity; *r* = −0.80), and raw scores are not directly comparable across dimensions because body morphology and fitness reflect relative population norms, whereas clinical health reflects absolute deduction thresholds. Future research should evaluate alternative data-driven weighting methods and consider alternative body composition indicators with less overlap. In addition, although the AHP panel included physicians, early childhood education teachers, and university researchers, parents, health visitors, kindergarten staff, and public health experts were not formally consulted. Future research should involve a broader range of stakeholders to improve content validity, acceptability, and practical relevance.

## 6. Conclusions

This study developed a physical health assessment system for preschool children, specified as a formative composite index of three dimensions (body morphology, physical fitness, and clinical health) and 13 indicators. Preliminary evidence supported its content coverage, indicator specification, absence of collinearity, and external criterion validity based on an independent health rating. However, these findings should be regarded as preliminary structural validity evidence, and national applicability requires further validation using samples from multiple regions.

## Figures and Tables

**Table 1:** Average pairwise comparison scores.

Dimension	Body Morphology	Physical Fitness	Clinical Health
Body Morphology	1.00	0.42	0.37
Physical Fitness	2.35	1.00	0.50
Clinical Health	2.68	1.99	1.00

**Table 2:** Normalized pairwise comparison results.

Dimension	Body Morphology	Physical Fitness	Clinical Health
Body Morphology	0.17	0.12	0.19
Physical Fitness	0.39	0.29	0.27
Clinical Health	0.44	0.58	0.53

**Table 3:** Consistency check.

Dimension	*A* *·* *w*	*Aw*/*w*	*λmax*
Body Morphology	0.49	3.02	3.03
Physical Fitness	0.96	3.04	
Clinical Health	1.59	3.05	

Note: *CI* = 0.02.

**Table 4:** Random consistency index (*RI*).

Order (n)	3	4	5	6	7	8	9
RI	0.52	0.89	1.12	1.26	1.36	1.41	1.46

**Table 5:** Deduction criteria for clinical health.

Indicator	Deduction Criteria
Dental caries	−2 points per decayed tooth, to a maximum of −10 points. Diagnostic criteria for dental caries follow the Technical standard for physical examination for students (GB/T 26343–2010). Each tooth was examined visually and with a probe. The number of decayed teeth was recorded for each child (Ministry of Health of the People’s Republic of China & Standardization Administration of China, 2011).
Overweight/obesity	Overweight: −10 points; obesity: −15 points. According to the Growth standard for children under 7 years of age (WS/T 423–2022), overweight and obesity were defined from BMI-for-age: overweight as a BMI-for-age between +1 SD and +2 SD of the reference population, and obesity as a BMI-for-age between +2 SD and +3 SD, with a BMI-for-age ≥ +3 SD classified as severe obesity (National Health Commission of the People’s Republic of China, 2022). The classification is norm-referenced.
Refractive error	Mild refractive error: −3 points; moderate: −5 points; severe: −10 points. Refractive abnormality was defined with reference to the Service specification for eye care and vision screening in children aged 0–6 years (trial) (No. 11 [2021] of the General Office of the National Health Commission; National Health Commission of the People’s Republic of China, 2021). At the participating hospital, the screening criteria were applied as follows: astigmatism ≥ 2.00 D, hyperopia ≥ +3.00 D, or myopia ≤ −1.50 D in either eye, or an inter-ocular difference in spherical power ≥ 2.00 D.
Malnutrition	stunting: −5 points; wasting: −5 points; underweight: −10 points. According to Growth standard for children under 7 years of age (WS/T 423–2022). Stunting was defined as height-for-age < −2 SD, wasting as weight-for-height < −2 SD, underweight as weight-for-age < −2 SD. The classification is norm-referenced.
Anaemia	Anaemia: −3 points. Following the Method for screening anaemia in populations (WS/T 441–2013), anaemia was defined as a haemoglobin concentration below 110 g/L; this threshold was applied to all children irrespective of age (National Health Commission of the People’s Republic of China, 2013).

**Table 6:** Weights of the physical health assessment system for preschool children.

First-Level Indicator	Weight	Second-Level Indicator	Weight
Body Morphology	0.163	Height	0.109
		BMI	0.054
Physical Fitness	0.317	Sit-and-reach	0.045
		Standing long jump	0.045
		Grip strength	0.045
		Two-foot continuous jump	0.068
		15 m obstacle run	0.045
		Balance-beam walking	0.068
Clinical Health	0.520	Dental caries	–
		Overweight/obesity	–
		Refractive error	–
		Malnutrition	–
		Anaemia	–

**Table 7:** Criterion-related validity of the physical health assessment index (*N* = 1462).

Criterion Item	Clinical Health	Body Morphology	Physical Fitness	Formative Index	Conventional Assessment
Healthier than most children	0.462 ***	0.321 ***	0.286 ***	0.596 ***	0.389 ***
Does not need to see a doctor	0.434 ***	0.292 ***	0.294 ***	0.570 ***	0.382 ***
Has reached expected health status	0.467 ***	0.332 ***	0.322 ***	0.622 ***	0.424 ***
Has fewer colds than other children	0.440 ***	0.312 ***	0.294 ***	0.581 ***	0.389 ***
Four-item scale	0.603 ***	0.421 ***	0.400 ***	0.793 ***	0.530 ***

Note: *** *p* < 0.001.

**Table 8:** Structural coefficients of the 13 formative indicators on the physical health index in the MIMIC model (*N* = 1462).

Dimension	Indicator	*γ*	SE	*p*
Clinical health	Anaemia	−0.028	0.019	0.139
	Refractive error	−0.064	0.021	0.002
	Dental caries	−0.449	0.020	<0.001
	Overweight/obesity	−0.386	0.033	<0.001
	Malnutrition	−0.163	0.026	<0.001
Body morphology	Height	0.342	0.023	<0.001
	BMI	0.144	0.031	<0.001
Physical fitness	Sit-and-reach	0.101	0.021	<0.001
	Standing long jump	0.094	0.025	<0.001
	Grip strength	0.074	0.020	<0.001
	Two-foot continuous jump	0.198	0.024	<0.001
	15 m obstacle run	0.056	0.022	0.012
	Balance-beam walking	0.210	0.023	<0.001

Note. *γ* = standardized structural coefficient from the indicator to the latent index. Negative coefficients denote clinical indicators coded so that higher values indicate worse health.

## Data Availability

The data presented in this study are available on request from the corresponding author. The data are not publicly available due to the privacy of the child participants and the sensitive health information involved.

## Supplementary Materials

The following supporting information can be downloaded at: https://www.mdpi.com/article/10.3390/bs16101925/s1, Table S1: Sensitivity of the MIMIC coefficients of the 13 indicators to alternative specifications and estimators (*N* = 1462); Table S2: Indicator-deletion sensitivity of the composite (*N* = 1462); Table S3: Sensitivity of the composite index to the scoring, weighting, deduction-value and indicator-deletion specifications (*N* = 1462).
