# Supplementary material for: Construction and Preliminary Validation of a Multidimensional Physical Health Assessment System for Preschool Children in China
Source: Behav Sci (Basel). 2026 Oct 17;16(10):1925. doi: 10.3390/bs16101925 (PMC13701325; doi:10.3390/bs16101925)
Supplement: Supplementary file 1 [file behavsci-16-01925-s001.zip › behavsci-4597843-supplementary.pdf]

Table S1. Sensitivity of the MIMIC coefficients of the 13 indicators to alternative specifications and estimators (*N* = 1,462).

Indicator	γ (WLSMV)	γ (binary)	Bootstrap 95% CI
A1 Anaemia	−0.027	−0.038	[−0.063, 0.010]
A2 Refractive error	−0.064	−0.068	[−0.108, −0.026]
A3 Dental caries	−0.455	−0.470	[−0.489, −0.413]
A4 Overweight/obesity	−0.382	−0.342	[−0.454, −0.315]
A5 Malnutrition	−0.160	−0.140	[−0.215, −0.115]
B1 Height	0.340	0.341	[0.295, 0.388]
B2 BMI	0.141	0.186	[0.083, 0.201]
C1 Sit-and-reach	0.104	0.099	[0.057, 0.140]
C2 Standing long jump	0.089	0.102	[0.047, 0.145]
C3 Grip strength	0.077	0.082	[0.032, 0.114]
C4 Two-foot continuous jump	0.197	0.193	[0.148, 0.243]
C5 15-m obstacle run	0.056	0.075	[0.014, 0.101]
C6 Balance-beam walking	0.211	0.203	[0.165, 0.256]

Note. γ = standardized structural coefficient. A1–A5, clinical health; B1–B2, body morphology; C1–C6, physical fitness. γ (WLSMV) was re-estimated treating reflective items as ordinal. γ (binary) was calculated after dichotomising clinical indicators (any vs. no deduction). Bootstrap 95% CIs are based on 1,000 resamples. Model fit: MLR $\chi^2(41) = 27.95$, $p = 0.940$, $R^2 = 0.762$; WLSMV $\chi^2(41) = 31.64$, $p = 0.853$, $R^2 = 0.756$; binary $\chi^2(41) = 37.43$, $p = 0.630$, $R^2 = 0.775$.

Table S2. Indicator-deletion sensitivity of the composite (*N* = 1,462)

Indicator deletion	Domain	Pearson *r* with baseline	Spearman ρ
Height	Body morphology	0.898	0.915
BMI		0.964	0.969
Overweight/obesity	Clinical health	0.888	0.895
Dental caries		0.820	0.787
Refractive error		0.995	0.996
Malnutrition		0.986	0.986
Anaemia		0.999	1.000
Sit-and-reach	Physical fitness	0.990	0.989
Standing long jump		0.995	0.994
Grip strength		0.991	0.990
Two-foot continuous jump		0.984	0.983

15-m obstacle run		0.995	0.995
Balance-beam walking		0.977	0.974
BMI + overweight/obesity	Body morphology + clinical health	0.768	0.785
Height + overweight/obesity		0.907	0.906

Note. Pearson r and Spearman ρ quantify the agreement between each deletion-based composite and the baseline composite.

Table S3. Sensitivity of the composite index to the scoring, weighting, deduction-value and indicator-deletion specifications (*N* = 1,462).

Specification	*r*	Reclassification (%)	*κ*	Not in lowest level (%)
Panel A. Scoring and scale				
Baseline (published specification)	1.000	0.0	1.000	84.5
Three dimensions z-standardized, AHP weights unchanged	0.996	5.3	0.947	84.6
Clinical dimension: linear mapping to 50–90	0.952	21.6	0.790	84.3
Clinical dimension: unmapped deduction score (70–100)	0.935	26.2	0.746	84.1
Clinical dimension: four-step mapping (60–90)	0.988	5.7	0.945	84.1
All 13 indicators min–max rescaled and equally weighted	0.906	32.7	0.684	83.8
Panel B. Weighting				
Baseline (AHP weights)	1.000	0.0	1.000	84.5
AHP weights recomputed by geometric-mean aggregation	1.000	0.3	0.997	84.5
Three dimensions equally weighted (original scales)	0.933	26.4	0.746	83.2
Three dimensions equally weighted (z-standardized)	0.964	19.6	0.812	83.4
Entropy weights	0.923	28.1	0.730	83.1
All 13 indicators equally weighted (original scales)	0.726	51.1	0.460	84.2
All 13 indicators equally weighted (z-standardized)	0.832	42.3	0.585	84.9
Panel C. Deduction values				
Baseline (k = 1.00)	1.000	0.0	1.000	84.4

k = 0 (clinical dimension excluded)	—	54.6	0.407	83.7
k = 0.25	—	27.1	0.739	83.2
k = 0.50	0.923	28.1	0.733	83.5
k = 0.75	—	18.0	0.828	84.0
k = 1.25	—	8.7	0.916	83.8
k = 1.50	—	22.7	0.785	83.4
k = 2.00	—	24.4	0.768	83.9
Deduction values re-derived from empirical criterion weights	0.854	34.7	0.648	84.1
Panel D. Indicator deletion (r and ρ for the same specifications are reported in Table S2)				
Baseline	—	0.0	1.000	84.5
Height	—	28.7	0.715	85.8
BMI	—	16.3	0.843	84.4
Overweight/obesity	—	21.1	0.775	83.7
Dental caries	—	50.0	0.509	84.2
Refractive error	—	2.0	0.981	84.5
Malnutrition	—	4.6	0.954	85.2
Anaemia	—	0.5	0.995	84.5
Sit-and-reach	—	8.8	0.915	84.1
Standing long jump	—	5.2	0.950	84.7
Grip strength	—	7.6	0.927	84.1
Two-foot consecutive jumps	—	11.4	0.890	84.8
15-m obstacle run	—	5.5	0.947	84.0
Walking the balance beam	—	16.5	0.840	85.3
BMI + overweight/obesity	—	32.2	0.634	83.8
Height + overweight/obesity	—	25.3	0.749	84.9

Note. The composite index is reported as a continuous score in the manuscript. The four-level grading (≥75.75; 68.08–75.75; 60.41–68.08; <60.41; based on sample mean ± 1 *SD*) was used only as an analytical criterion for sensitivity testing. Reclassification = percentage of children shifting levels relative to baseline; κ = linear-weighted Cohen's κ; "% not in lowest level" = percentage of children with composite score ≥60.41. Panel A: alternative clinical scoring or dimension scaling. Panel B: AHP weights replaced or re-aggregated. Panel C: k scales all five deduction values. Panel D: deleted indicators' weights redistributed proportionally within their dimension. Across all specifications, the proportion not in the lowest level remained stable (83.1%–85.8%), whereas finer level assignment was more sensitive (reclassification: 0.3%–54.6%). *N* = 1,462.
